# Bazooka mediates secondary axon morphology in *Drosophila *brain lineages

**DOI:** 10.1186/1749-8104-6-16

**Published:** 2011-04-27

**Authors:** Shana R Spindler, Volker Hartenstein

**Affiliations:** 1 Department of Molecular Cell and Developmental Biology, University of California Los Angeles, Los Angeles, CA 90095, USA

## Abstract

In the *Drosophila *brain, neural lineages project bundled axon tracts into a central neuropile. Each lineage exhibits a stereotypical branching pattern and trajectory, which distinguish it from other lineages. In this study, we used a multilineage approach to explore the neural function of the Par-complex member Par3/Bazooka *in vivo*. *Drosophila bazooka *is expressed in post-mitotic neurons of the larval brain and localizes within neurons in a lineage-dependent manner. The fact that multiple GAL4 drivers have been mapped to several lineages of the *Drosophila *brain enables investigation of the role of Bazooka from larval to adult stages *Bazooka *loss-of-function (LOF) clones had abnormal morphologies, including aberrant pathway choice of ventral projection neurons in the BAla1 lineage, ectopic branching in the DALv2 and BAmv1 lineages, and excess BLD5 lineage axon projections in the optic medulla. Exogenous expression of Bazooka protein in BAla1 neurons rescued defective guidance, supporting an intrinsic requirement for Bazooka in the post-mitotic neuron. Elimination of the Par-complex member Par6 recapitulated Bazooka phenotypes in some but not all lineages, suggesting that the Par complex functions in a lineage-dependent manner, and that Bazooka may act independently in some lineages. Importantly, this study highlights the potential of using a multilineage approach when studying gene function during neural development in *Drosophila*.

## Background

Neurons of the *Drosophila *brain are grouped into individual units, termed lineages. All neurons belonging to a single lineage are derived from a common neuroblast. Neurons born in the embryo and larva compose the primary and secondary lineages, respectively. Each group of secondary neurons emits a secondary axon tract (SAT) into the central neuropile along the existing primary axon tracts (PATs). As early as embryonic development, lineages begin to acquire a unique morphology that is retained into the larval stages [1-3]. During pupation, approximately 40% of embryonic-born primary neurons are lost, and the secondary neurons begin to generate an intricate network of arbors in the pupal neuropile compartments [3]. SATs are the primary scaffolding and functional units of the adult brain, therefore in the context of using *Drosophila *as a model to understand circuit formation, it is important to elucidate the mechanisms that underlie stereotypical lineage morphologies.

Key aspects of neuron growth that dictate morphology include axon guidance, branch formation and axon versus dendrite specification. Previous reports indicate a role for the Par-complex proteins (Par3, Par6 and atypical protein kinase (aPK)C) during the latter. Cultured mammalian hippocampal neurons initially send out numerous processes, which all have equal potential to become dendrites or axons. The neurite that becomes the axon retains high levels of Par proteins at its tip. Ectopic expression of Par3 or Par6 or inhibition of aPKC results in neurons which lack a single specified axon [4,5]. There are two possible mechanisms by which the Par complex affects axon selection from a pool of neurites. On the one hand, the Par complex may segregate additional axon-specific proteins into a single neurite, whereas on the other hand, growth-cone accumulation of Par-complex members could enable one neurite to competitively outgrow other neurites to win axon fate. The axon-specific machinery affected by ectopic expression of Par-complex members is currently unknown.

**Figure 1:**
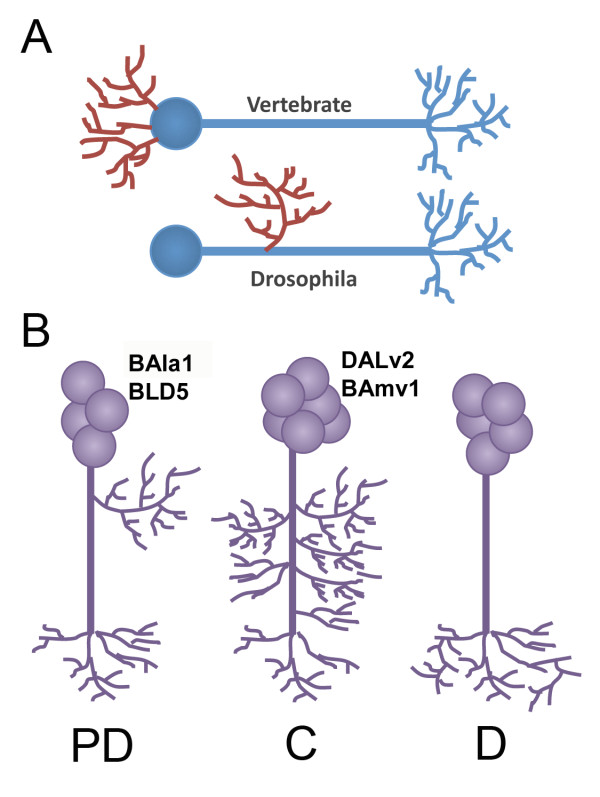
***Drosophila *neuron morphology**. **(A) **Cartoon depiction of a bipolar vertebrate neuron emitting dendrites (red) from the cell body compared with a *Drosophila *central nervous system (CNS) neuron containing collateral dendrites. **(B) **The three morphologic categories of Drosophila lineages: proximal (PD) lineages contain a distinct domain of proximal branching physically separated from a set of terminal branches, distal (D) lineages have only distal branching, and continuous (C) lineages have wide domains of collateral branches along most of the axon fascicle. Lineages used in this study are indicated next to each lineage type

In *Drosophila *and other insects, neurons are unipolar, with postsynaptic dendritic terminal fibers and presynaptic axonal fibers branching off a single main neurite (Figure 1A) [6]. The dendritic patterning varies between lineages of *Drosophila *neurons: proximal-distal (PD) lineages include distinct domains of dendritic proximal branching (such as those seen in the calyx of the mushroom body), whereas distal (D) and continuous (C) lineages have mixed axonal and dendritic domains ([3]; Figure 1B).

Elimination of the Par complex from the *Drosophila *mushroom body does not disrupt the formation of dendrites and axons [7]. This suggests that either 1) *Drosophila *brains use a separate mechanism for axon and dendrite specification because of the unique organization of the neuron; 2) *in vitro *mammalian studies do not reflect the actual cellular events which take place *in vivo*; or 3) the Par complex controls a cellular process (such as axon guidance or branching) that may differ between neurons in the *Drosophila *brain. We suspect the third possibility to be correct, and localization of Par3 to the tip of the extending axon in cultured hippocampal cells makes it tempting to speculate a role for the Par complex in the growth cone during neurite growth and guidance.

Par-complex proteins have a rich history in invertebrate models. These polarity proteins were originally identified in *Caenorhabditis. elegans *as factors establishing the first asymmetric divisions that produce the anterior and posterior cells [8]. During *Drosophila *embryonic development, the Par complex has a well-described role in epithelial polarity [9-13]. Bazooka, the *Drosophila *homolog of Par3, is present at the apical basal boundary in adherens junctions even before the arrival of *Drosophila *E-cadherin [14]. Null mutations in *bazooka *result in embryonic lethality, in which large holes form in the cuticle (hence the name *bazooka*) and embryos fail to undergo dorsal closure [15].

In the *Drosophila *brain, the Par complex is traditionally associated with asymmetric division of the neuroblast [16] and spindle orientation during neuroblast division [11]. Whereas Bazooka is actively sequestered from the daughter ganglion mother cell (GMC) during neuroblast division, it is re-expressed in the post-mitotic neurons of at least the mushroom body [13]. However, it has not been reported whether Bazooka is globally re-expressed in the transient GMC or post-mitotic neurons throughout the *Drosophila *brain.

In addition to regulating cellular polarity and asymmetric divisions, the polarity proteins have also been studied in the context of directional guidance. *In vitro*, aPKC and Par3 localize to the leading edge of scraped Madin-Darby canine kidney (MDCK)11 cells under the control of PATJ (protein associated with Lin 7 (PALS)1-associated tight junction) during wound closure [17]. Par3 and T-cell lymphoma invasion and metastasis (Tiam)1 are required at the leading edge of freely migrating polarized keratinocytes for directional guidance [18]. Finally, phosphorylated Par3 protein modulates front-rear polarity from the leading edge of migrating Hela cells [19]. *In vivo*, perturbation of the Par complex affects directional orientation of the leading process in rhombic lip-cell migration in the developing chicken cerebellum [20], and it is required for border-cell migration in *Drosophila *embryos [21].

The establishment of apical-basal polarity, spindle orientation and directional migration all require strict control of the cytoskeletal machinery. This array of functions controlled by the Par complex can potentially be explained by its ability to associate with cytoskeletal regulators, such as Rac1, a common denominator in many cellular events [22,23]. We speculate that ectopic Par-complex expression in cultured mammalian neurons disrupts the cytoskeletal machinery used during neurite outgrowth, therefore inhibiting axon-dendrite formation. Whether polarity proteins are required for growth-cone movement or guidance, as suggested by their localization to the growth cone in culture conditions[5], has yet to be reported.

What *in vivo *role does the Par complex command during axon outgrowth? *Drosophila *neural lineages present an advantageous model to address this question. Lineages are relatively large, and have stereotyped morphologies that can be followed throughout development. At third instar, SATs have an unbranched trajectory into the central neuropile, which can be identified according to established nomenclature [24]. During pupal stages, each lineage expands and elaborates via pathfinding, branching and pruning to produce the final lineage morphologies of the adult brain.

To investigate the *in vivo *role of Par-complex proteins during neural growth and guidance, we generated *bazooka *LOF clones with the help of mosaic analysis with a repressible cell marker (MARCM) and lineage-specific drivers [25]. We found that Bazooka is re-expressed in post-mitotic neurons, accumulates within the larval growth cone, and mediates directional guidance, branching and axon projection number in a lineage-dependent manner. We observe similar phenotypes upon manipulation of an additional Par-complex member, Par6. Based on this evidence, we propose a model in which Bazooka is utilized during axon growth to control diverse aspects of neural morphology, such as axon pathfinding and branching, depending on the shape each neuron must acquire.

## Methods

### Markers and stocks

The larval secondary neurons and axon tracts were labeled with an antibody against neurotactin (BP106; Developmental Studies Hybridoma Bank, University of Iowa, IO, USA) and adult axon tracts were labeled with an antibody against neuroglian (BP104; Developmental Studies Hybridoma Bank). An antibody against *Drosophila *N-cadherin (DN-Ex#7, Developmental Studies Hybridoma Bank) was used to mark the neuropile in both larva and adult. The fly stocks used in this study were: UAS-Baz:GFP (with an upstream activator sequence (UAS) and a green fluorescent protein (GFP) tag; a gift from Dr Prokop) [6], UAS-Baz (a gift from Dr Doe) [7], UAS-mcd8:GFP (#5137; Bloomington Drosophila Stock Center, University of Indiana, IN, USA), *baz^4^*/FM7a (#3295l; Bloomington Drosophila Stock Center). To generate MARCM clones, we used the following stocks: FRT19A,hsFLP,tubGAL80;pins/Cyo (#5133; Bloomington Drosophila Stock Center), *period*-GAL4,UAS-GFP [26] and *atonal*-GAL4,UAS-GFP [27]. The following Flippase recognition target (FRT) lines were gifts from the Doe lab: FRT19A, FRT19A, *baz*^4^/FM7C and FRT19A, *par6*^Δ226^/FM7C [7].

### Immunohistochemistry and histology

The following antibodies and dilutions were used: anti-neurotactin 1:10 (BP106), anti-DN-Cadherin 1:20 (DN-Ex#7) (both Developmental Studies Hybridoma Bank); anti-Bazooka rabbit 1:500 (a gift from Dr Wodarz); Alexa 546-conjugated anti-rat Ig 1:500 (A11081), Alexa 546-conjugated anti-mouse Ig 1:500 (A11030) (both Molecular Probes, Eugene, OR, USA); and Cy5-conjugated anti-rat Ig 1:100 (112-175-102), FITC-conjugated anti-mouse 1:200 (115-095-100), Cy3-conjugated anti-rabbit Ig 1:200 (711-165-152) and Cy5-conjugated anti-mouse Ig 1:100 (115-175-166) (all Jackson ImmunoResearch, West Grove, PA, USA). For antibody labeling, standard procedures were followed [28]. In brief, brains were dissected in 1× phosphate-buffered saline (PBS) pH 7.4 and fixed in 4% formaldehyde 30 minutes at room temperature or overnight at 4°C. Fixed brains were washed with 1× PBS at least 30 minutes, and either stored in methanol at -20°C or further processed. After at least three washes of 15 minutes each in 1× PBS with Triton X-100 0.1 to 0.3% (PBT), brains were incubated in 10% normal goat serum (GS0500; Capralogics Inc., Hardwick, MA, USA) for at least 1 hour at room temperature, followed by primary antibody incubation overnight at 4°C. Brains were then washed at least 30 minutes in 1× PBT three times, incubated again in 10% normal goat serum for at least 1 hour, and left in secondary antibody for 2 hours at room temperature or overnight at 4°C. Finally, brains were washed at least four times for 15 minutes each in 1× PBT, and mounted on glass slides in mounting medium (Vectashield; H-1000; Vector Laboratories, Burlingame, CA, USA). Brains were viewed under a confocal microscope (20× or 40× objective; MRC 1024ES microscope with Radiance 2000 and Laser Sharp 2000, version 5.2 build 824 software; all Bio-Rad, Hercules, CA, USA). Confocal sections were taken at 2 μm intervals for all preparations. We used Imagej1.41d software (National Institutes for Health, Bethesda, MD, USA) for image analysis and for generation of merged confocal sections. In cases where confocal stacks contained overlapping SATs along the *Z*-axis, Imagej4.1 was used to mask non-target lineages before generating a merged stack.

### Clonal analysis

To visualize individual SATs in the adult brain, we generated MARCM clones [25] and visualized clones with lineage-specific driver lines *period *(*per*)-GAL4 and *atonal (ato)*-GAL4. To visualize clones in *period*- and *atonal*-expressing lineages, we generated flies with the following genotypes:

FRT19A,hsFLP,GAL80ts; *per-*GAL4,UASGFP/CyO

FRT19A,hsFLP,GAL80ts; *per-*GAL4,UASGFP/Cyo; UAS-Baz/TM3; FRT19A,hsFLP,GAL80ts; *ato-*GAL4,UASGFP/CyO.

For controls, we crossed FRT19A virgin females with males of each of the above lines. For mutant clones, we crossed FRT19A, *baz*^4^/FM7C or FRT19A, *par6*^D226^/FM7C virgin females to males of the above lines. Only female progeny were used in our analysis.

Embryos were collected overnight on standard agar plates with yeast paste. Within 24 hours of hatching, first instar larvae were hand-picked, placed on standard fly food plates, and heat shocked for 1 to 2 hours at 37°C. After heat shock, the larvae were transferred to bottles with standard fly food and left at room temperature until eclosion. Female adult brains were dissected within 10 days of eclosion.

### Counting cell number in MARCM clones

In clones visualized with *atonal-*GAL4, cell numbers were counted in two ways: first, the volume of the clustered neurons was measured using Labelfield, SurfaceGen and Volume modules of Amira software (version 3.1.1; Mercury Computer Systems, Inc., Chelmsford, MA, USA). The total number was divided by 125 μm^3^, the measured volume of a single cell body. To verify the accuracy of the volume method, another set of *atonal-*GAL4 clones was generated and labeled with SYTOX blue (Invitrogen, Carlsbad, CA, USA) to manually count the cell body number with ImageJ1.41d. Both methods produced a similar number of cell bodies for their respective genotypes (data not shown).

## Results

### Bazooka is expressed in neuroblasts, GMCs and post-mitotic neurons in third instar larvae

**Figure 2:**
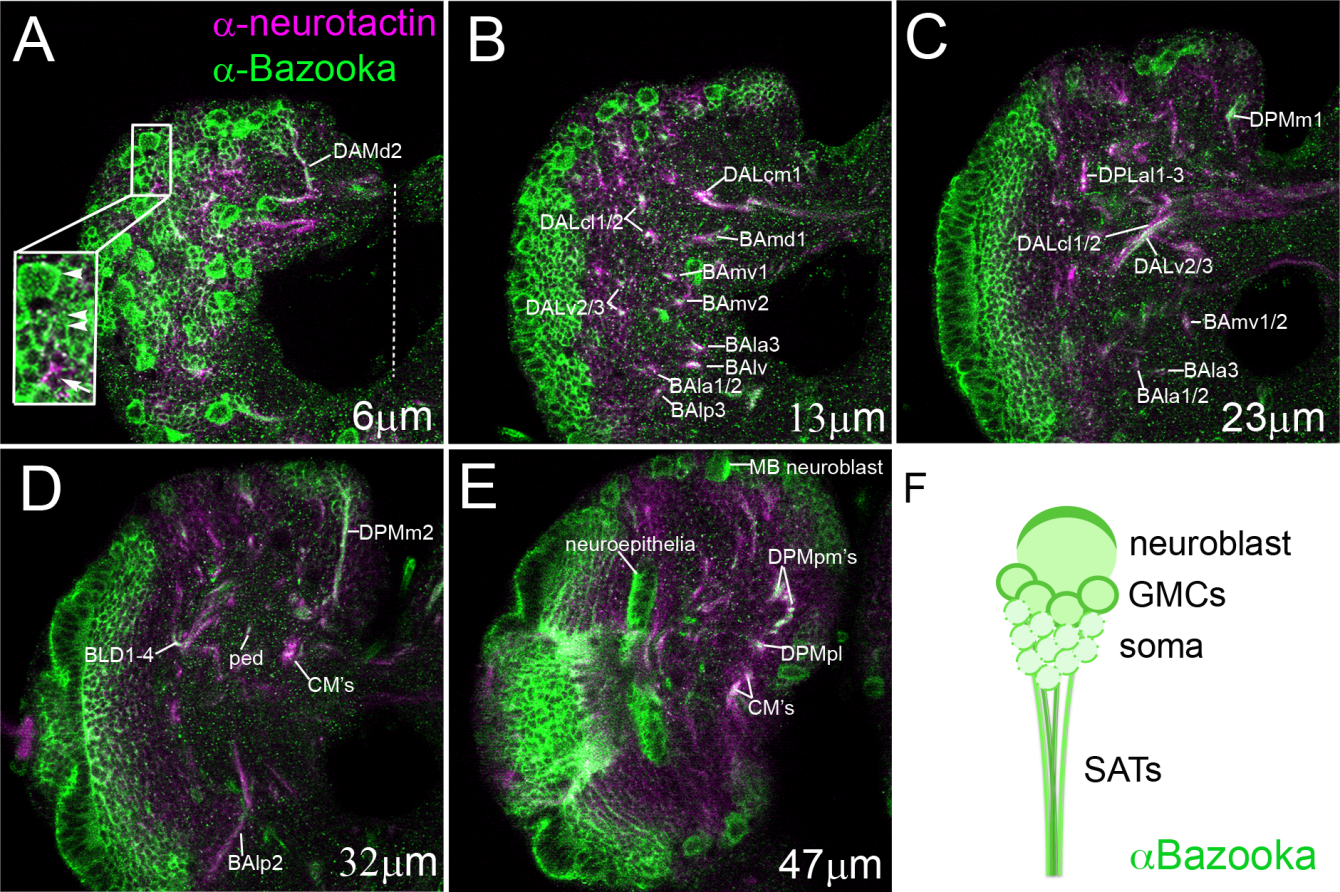
**Endogenous Bazooka protein expression at third instar**. **(A-E) **Bazooka expression visualized with a rabbit anti-Bazooka monoclonal antibody [39] and shown at varying distances into the brain (distance indicated in lower right in each panel) from anterior to posterior in 2 μm sections. Secondary axon tracts (SATs) are labeled according to published nomenclature [24]. (A) Magnified lineage shows Bazooka expression in the neuroblast (arrowhead), ganglion mother cells (GMCs) and newborn neurons (double arrowheads), and older neurons (arrow). (E) Note the apical accumulation of Bazooka protein in the apical mushroom body (MB) neuroblast and neuroepithelia (lateral is shown to the left and medial is to the right, with midline indicated by a dotted line). **(F) **A cartoon depiction of Bazooka localization (green) in the apical neuroblast, newly born neurons and to a lesser extent late-born neurons, and in the bundled axon tracts. Scale bar: 25 μm

To establish a global view of endogenous Bazooka localization throughout larval secondary lineages, we double-labeled third instar brains with monoclonal antibodies against Bazooka and Neurotactin, a surface glycoprotein that marks all SATs [24]. We sampled 2 μm thick slices from anterior, central and posterior positions in the brain. Bazooka was localized to the expected apical neuroblasts and apical neuroepithelia (Figure 2E). In addition, Bazooka was expressed and heavily localized to the membrane of GMCs and their most recent offspring (Figure 2A, arrowheads), and to a lesser extent, older neurons. Moving from anterior to posterior sections, Bazooka was visualized in most SATs as seen by the co-expression of Bazooka and neurotactin throughout the larval brain (Figure 2A-E, labeled SATs). Therefore, we conclude that Bazooka is re-expressed in the post-mitotic neuron and travels into the axon tracts of most, if not all, lineages (Figure 2F).

### Lineage-specific Bazooka:GFP localization

**Figure 3:**
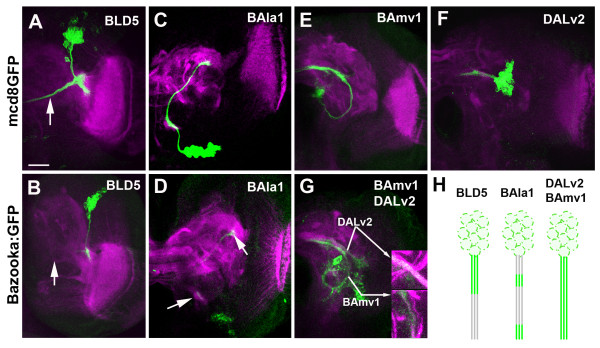
**Lineage-specific accumulation of Bazooka:green fluorescent protein (Baz:GFP)**. Third instar brains expressing mcd8GFP (top row) or Bazooka:GFP (bottom row) under the control of **(A,B) ***atonal-*GAL4 **(C-G) **or *period-*GAL4 and stained with anti-*Drosophila *N-cadherin (magenta). One hemisphere of each is shown. (A) *atonal-*GAL4>UAS-mcd8GFP expression in the BLD5 lineage showing tract projection toward the midline (arrow). (B) *atonal-*GAL4>UAS-Baz:GFP expression showing Baz:GFP sequestration to the proximal axon domain; no Baz:GFP is visualized in the medial projecting portion of the tract (arrow). (C) Mosaic analysis with a repressible cell marker (MARCM) clone in the BAla1 lineage with *period-*GAL4>UAS-mcd8GFP visualization. (D) *period-*GAL4>UAS-Baz:GFP expression showing Baz:GFP accumulation at a small proximal domain along the SAT and at the distal growth cone (arrows). (E,F) MARCM clone in (E) the BAmv1 and (F) DALv2 lineages visualized with *period-*GAL4>UAS-mcd8GFP. **(F) ***period-*GAL4>UAS-Baz:GFP expression showing Baz:GFP continuous expression throughout the BAmv1 and DALv2 SATs. **(H) **Cartoon depicting the various types of Baz:GFP (green) accumulation seen across the different lineages. Scale bar: 25 μm

Lineage-specific drivers were used to visualize a small number of lineages in the brain. For the remainder of this study, we used *period-*GAL4 (labeling BAla1, BAmv1 and DALv2) and *atonal-*GAL4 (labeling BLD5). Using each lineage-specific GAL4 driver, we expressed a previously characterized Bazooka:GFP (Baz:GFP [6]) fusion protein to determine if Bazooka accumulates equally in all lineages. In previous studies it was confirmed that expression of this fusion protein mirrored the expression of the endogenous protein (for example,, [6,29]). We found that Baz:GFP accumulated in unique domains of each SAT of the various lineages (Figure 3H), and remained in the proximal region of the BLD5 lineage (Figure 3A,B). Owing to overlap between the ipsilateral/proximal elbow with the contralateral/distal end, we could not exclude Baz:GFP localization to the distal growth cone in the BLD5 lineage. In the BAla1 lineage, Baz:GFP accumulated in the cell bodies, in a small tuft of filopodia along the proximal SAT, and at the terminal growth cone (Figure 3C,D). Baz:GFP was continuously found along the axon tracts in both the BAmv1 and DALv2 lineages (Figure 3E-G). It is noteworthy that a correlation emerged when Baz:GFP localization was compared with PD- or C-type lineage patterning. Thus, in the PD-type lineages BAla1 and BLD5, Baz:GFP accumulated in specific domains of the axon, in particular at the location of future proximal branches. In the non-PD-type lineages (BAmv1 and DALv2), Baz:GFP seemed to have an even distribution along the SAT fascicles.

### Developmental profile of wild-type BAla1, DALv2 and BAmv1

Srahna and colleagues previously reported a developmental series for the BLD5 lineage [30]. The presence of a distinct point of proximal branches sequestered from distal arbors distinguishes BLD5 as a PD-type lineage. In this study, we established the developmental profile for the *period-*GAL4 expressing lineages BAla1, BAmv1 and DALv2.

The BAla1 lineage contains the multiglomerular projection neurons of the antennal lobe (AL) [31]. In the third instar stage, cell bodies form a cluster in the anterior cortex, ventrally adjacent to the basal anterior (BA) compartment. The BAla1 SAT projected dorsoposteriorly through the middle antennal cerebral tract (mACT) in the larval brain, ultimately terminating in the centroposterior lateral (CPL) compartment. A small tuft of filopodia marked the location of future branches on the proximal SAT (see Additional file 1, Figure S1A). In pupal stages, proximal neurites branched into the presumptive AL, and the terminal SAT grew into the lateral horn (see Additional file 1, Figure S1B). By eclosion, the BAla1 SAT projected widespread dendritic arborizations into the AL. In the posterior direction, the SAT continued as an unbranched lateral bundle in the mACT, and terminated in the lateral horn (see Additional file 1, Figure S1C). Because BAla1 has proximal branches in the AL and distal branches in the lateral horn, it is a PD-type lineage.

Neurons of the DALv2 lineage innervate most of the ellipsoid body. At third instar, the DALv2 SAT projected dorsomedially into the centroposterior medial (CPM) compartment [21] (see Additional file 1, Figure S1D). During pupation, the SAT emitted proximal branches into the glomeruli of the developing lateral bulb (the major input domain of the ellipsoid body), and distal branches into the midline of the brain at the position of the presumptive ellipsoid body (see Additional file 1, Figure S1E). At eclosion, this lineage had full proximal branches that stretched dorsally into the inferior-medial protocerebrum (IMP), ventrally into the lateral accessory lobe (LAL) and additionally into the lateral bulb (LB). Finally, the terminal arbors branched throughout multiple layers of the ellipsoid body (see Additional file 1, Figure S1F). Because the DALv2 lineage has a large domain of branches along the SAT projecting into the LB, IMP and LAL, we consider it a C-type lineage.

The BAmv1 lineages contribute to the adult fan-shaped body and LAL. Initially, the larval BAmv1 SAT projected ventrally into the neuropile and twisted dorsoposteriorly into the longitudinal basomedial fascicle (loBM) [24]. Like the BAla1 SAT, small tufts of filopodia marked points of future branching (see Additional file 1, Figure S1G). During pupal stages, distal branches began to form the fan-shaped body at the midline (see Additional file 1, Figure S1H), which was complete in the adult brain (see Additional file 1, Figure S1I), and a set of finer continuous branches were projected into the LAL and inferior protocerebrum. The continuous branch pattern along approximately half of the BAmv1 adult SAT is indicative of a C-type lineage.

### Bazooka elimination disrupts SAT morphology

**Figure 4:**
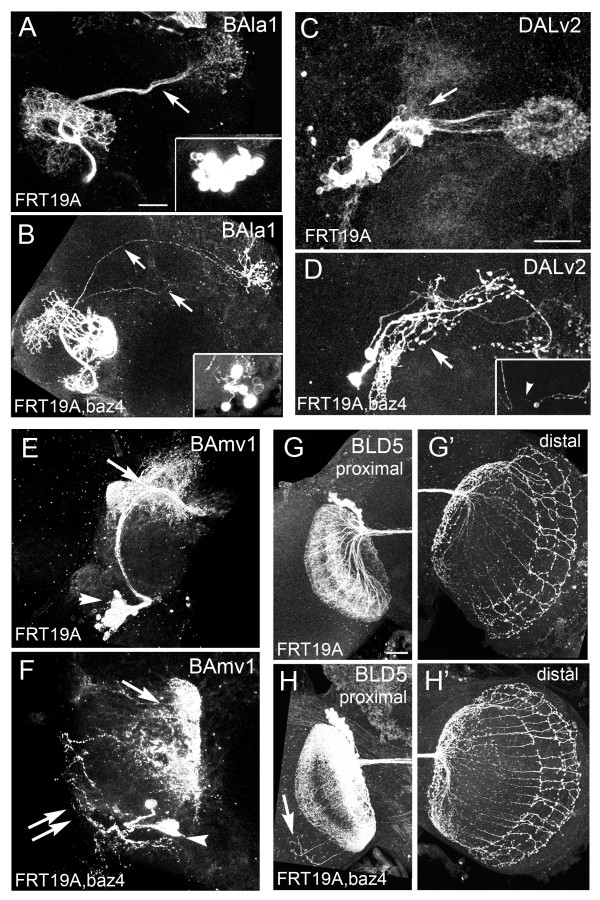
**Bazooka loss-of-function (LOF) clones in multiple lineages**. Adult hemispheres showing *Bazooka *LOF MARCM clones induced at first instar and visualized with either **(A-F) ***period*-GAL4 **(G,H) **or *atonal*-GAL4. (A,B) Mosaic analysis with a repressible cell marker (MARCM) clones in the ventral projection neurons (vPNs) of the BAla1 lineage visualized with *period-*GAL4,UASGFP. (A) Control BAla1 secondary axon tracts only entered the middle antennal cerebral tract (mACT) (arrow). (B) BAla1 clones containing null alleles of *bazooka *were misguided into the mACT and inner (i)ACT (arrows). (C) control DALv2 clone with proximal arbors in the lateral triangle and IMP (arrow) and distal arbors in the central ellipsoid body (EB). (D) *baz *LOF clone in the DALv2 lineage with ectopic bulbous proximal projections (arrow). Inset shows a second DALv2 clone exhibiting a long bipolar-like neurite (arrowhead). **(E) **Control BAmv1 clone with distal projections into the fan-shaped body and inferior-medial protocerebrum (IMP) (arrow). (F) *baz *LOF clone with disorganized branching along the axon tract (double arrows) and abnormal distal arbors (arrow). Note smaller clone size in all *baz *LOF clones visualized with the *period*-GAL4 driver (arrowheads mark cell bodies). (G,G') BLD5 control clone visualized with *atonal*-GAL4 showing proximal projections into (G) the ipsilateral optic lobula and (G') about 11 distal branches in the contralateral optic medulla. (H,H') *Baz *LOF BLD5 clone visualized with *atonal*-GAL4 showing (H) ectopic projections into the ipsilateral medulla and (H') increased axonal extensions in the contralateral optic medulla. In all preparations, a single hemisphere is shown of an adult brain. Scale bars: 25 μm

Using the developmental profiles of the BAla1, BAmv1, DALv2 and BLD5 lineages, we next investigated the *in vivo *function of Bazooka protein in neurons. To this end, we induced *bazooka *LOF MARCM clones at first instar, and visualized recombination events with the *period*-GAL4 and *atonal*-GAL4 drivers in adult brains. Figure 4 shows *bazooka *LOF and control clones for each lineage in one hemisphere of the adult brain; each phenotype is described in detail below.

In the process of screening through hundreds of brains, we found only one clone in the BAmv1 lineage and two clones in the DALv2 lineages, suggesting that *bazooka *loss of function in these particular lineages inhibits *period*-GAL4-controlled GFP expression, or that these lineages require Bazooka for growth and maintenance. Although only a small number of clones were obtained, it is noteworthy that both the C-type BAmv1 and DALv2 SATs displayed a large amount of branching along the length of the tract (Figure 4F,D). Control clones in these lineages never exhibited such branching patterns. In control DALv2 clones, all branching in the proximal DALv2 SAT had normal morphology (Figure 4C, arrow). By contrast, the ectopic branches in the *bazooka *LOF DALv2 clones had a bulbous morphology resembling axonal terminals (Figure 4D).

We next examined BLD5 SAT morphology in control and *bazooka *LOF clones. The BLD5 lineage consists of approximately 40 neurons, also known as dorsal cluster neurons [30]. It has been shown previously that in the adult, BLD5 neurons project proximal arbors into the lobula of the ipsilateral optic lobe and a stereotypical set of axon projections into the contralateral optic medulla. At mid-pupa, the distal axon projections into the medulla number approximately 30, whereas only 11 axons remain in the medulla by eclosion [30]. Similar to these previous reports, our control clones averaged 11 axon projections into the medulla (Figure 4G', n = 23). However, *bazooka *LOF clones averaged approximately 17 distal axons (Figure 4H', n = 14), suggesting that either too many axons are initially projected or that too few axons are retracted from the medulla during pupal development. In addition to ectopic terminal projections, we also saw an increase in the number of proximal projections (Figure 4G,H). We never found more than one extension into the ipsilateral medulla in the control clones, whereas two or more proximal projections into the medulla were seen in the *bazooka *LOF clones (Figure 4H).

The last lineage we analyzed was BAla1. In control BAla1 clones, 100% of the clones remained bundled as a single fascicle in the mACT (Figure 4A). By contrast, 91% (n = 11) of the *bazooka *LOF clones reached the lateral horn by way of the inner (i)ACT, a tract system normally taken by the dorsal and lateral projection neurons that form part of three other (*period-*negative) lineages [31] (Figure 4B). Finally, the *bazooka *LOF clones regularly contained three to five neurons, whereas control clones contained 20 or more cells. The visualization of a few neurons per clone is most likely due to misregulation of the *period-*GAL4 driver rather than to general defects in division or neuron survival, because a small clone size was seen only in *bazooka *LOF clones visualized with *period-*GAL4, but not with *atonal-*GAL4 (Figure 4H) or *elav-*GAL4 drivers (data not shown). However, we cannot exclude that *bazooka *affects cell proliferation/survival in the specific subset of lineages that are visualized with *period*-GAL4.

In summary, the C-type lineages had ectopic branching patterns whereas the two PD-type lineages had ectopic axon guidance/retraction phenotypes in *bazooka *LOF clones.

### Bazooka is not required for establishment of dendrite *versus *axon in the BAla1 lineage

**Figure 5:**
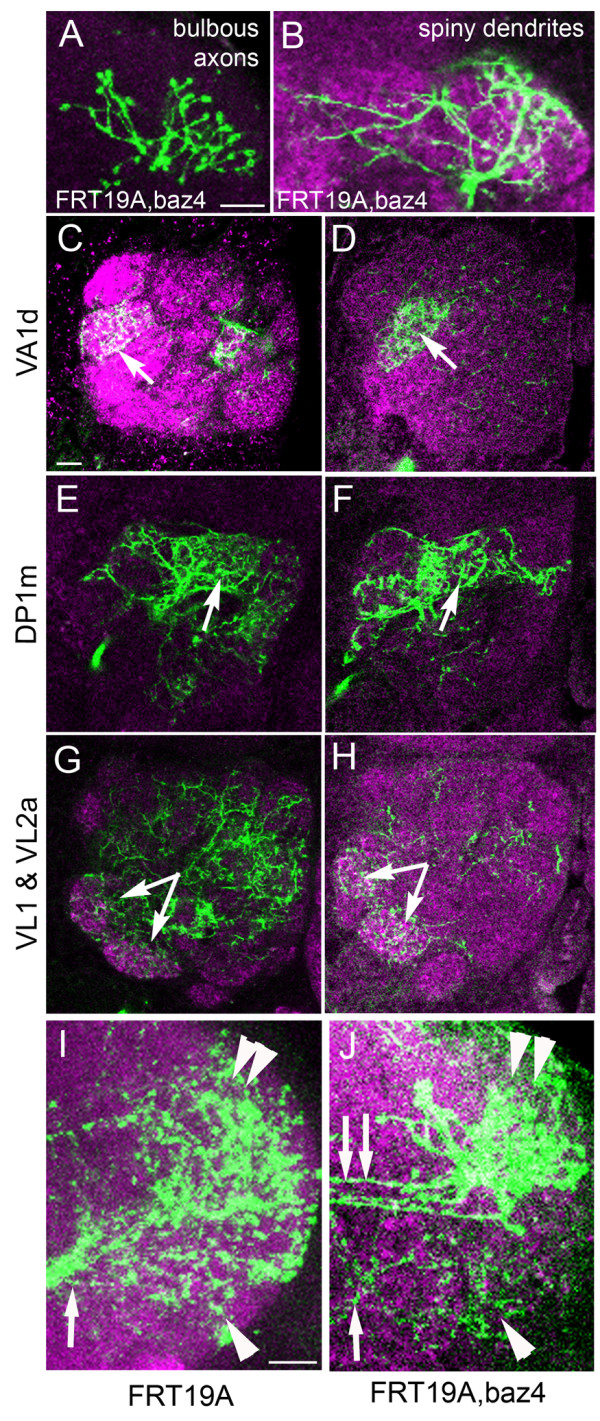
**BAla1 clones lacking Bazooka retain dendrite and axon morphology**. **(A) **Ventral projection neuron (vPN) terminal branches retained bulbous morphology in an adult preparation of a *baz *loss-of-function (LOF) BAla1 clone. **(B) **vPN proximal branches in the glomeruli of the antennal lobe exhibited typical postsynaptic spiny morphology. **(C,E,G) **The typical pattern of glomeruli infiltration by the proximal branches of the ventral projection neurons was retained in **(D,F,H) **clones containing the *baz*^4 ^null allele. The glomeruli innervated are indicated to the left of each row. **(I) **Terminal branching of a control BAla1 clone into the lateral horn. Note all branching stems from a single fascicle (arrow) and branching reached into both the dorsal (double arrowhead) and ventral (arrowhead) lateral horn. **(J) **Terminal branching of a *baz *LOF BAla1 clone into the lateral horn. Note that branching stemmed from both a ventral fascicle (arrow) and a set of dorsal fasicles (double arrows), and that the lateral horn area innervated correlated with the point of fascicle entry (dorsal innervation with dorsal entry, double arrowhead; ventral innervation with ventral entry, arrowhead). The neuropile was labeled with anti-Drosophila N-cadherin in all panels (magenta). Scale bars: 10 μm

Cultured mammalian hippocampal neurons ectopically expressing Par3 fail to polarize into axons and dendrites [5]; however, elimination of Bazooka in the *Drosophila *mushroom bodies does not disrupt neuron morphology [7]. Is this also the case for *Drosophila *projection neurons? We next examined if atypical guidance of the BAla1 SAT is due to a mis-specification of axons and dendrites. We first characterized proximal and terminal arbor morphology in the *bazooka *LOF clones. The proximal branches in *bazooka *LOF clones exhibited a 'spiny' morphology containing thin terminal protrusions, whereas the distal branches had a 'bulbous' morphology reminiscent of axon endings (Figure 5A,B).

We next investigated whether the dendrites targeted appropriately. The wild-type proximal arbors of the BAla1 lineage were multiglomerular in the AL, but some glomeruli were innervated more densely than others. Many of the VA1d and DP1m glomeruli were extensively filled with BAla1 proximal branches, whereas most of the VL1 and VL2a glomeruli were only sparsely filled (Figure 5C,E,G). Even with fewer neurons visualized, the *bazooka *LOF clones continued to project into the same glomeruli as control clones, but more densely than controls (Figure 5D,F,H; see Additional file 2, FigureS2), suggesting that not only are dendrites specified, but they also retain their same character. We did observe a few glomeruli that were innervated more often in the *bazooka *LOF clones than in the controls. The DM1, DP1l and VL2a glomeruli contained dense arborizations in at least 20% of the *bazooka *LOF clones, whereas the DA4, DL5, VA1lm, VL2p, VM7, VP1-3 and SOG glomeruli had increased arbors in approximately 10% of the *bazooka *LOF clones (see Additional file 2, Figure S2). However, it was difficult to determine if this innervation pattern is obscured in the wild types by the multiglomerular projections more common to control clones.

In the distal SAT, terminal arbors of the mutant BAla1 lineage branched into the correct vicinity of the lateral horn compared with control clones (Figure 5I,J), even though branches into the dorsal lateral horn derived from axons that traveled into the iACT. In the BLD5 lineage, both proximal and distal projections in *bazooka *LOF clones obeyed medullar boundaries similar to the control clones (see Figure 4G-H). Taken together, the data suggest that neurites with dendritic and axonal morphology are generated at their normal positions in BAla1 and BLD5 *bazooka *LOF clones. By contrast, the DALv2 clones obtained had ectopic proximal projections with bulbous morphology, resembling axonal endings (Figure 4D, arrow). It is noteworthy that both BAla1 and BLD5 lineages are PD-type, whereas the DALv2 lineage is C-type. Therefore, we cannot eliminate the possibility that *bazooka *is required differently by different lineage types in the establishment of dendrite versus axon.

### Baz is required intrinsically for axon guidance of the BAla1 SAT

**Figure 6:**
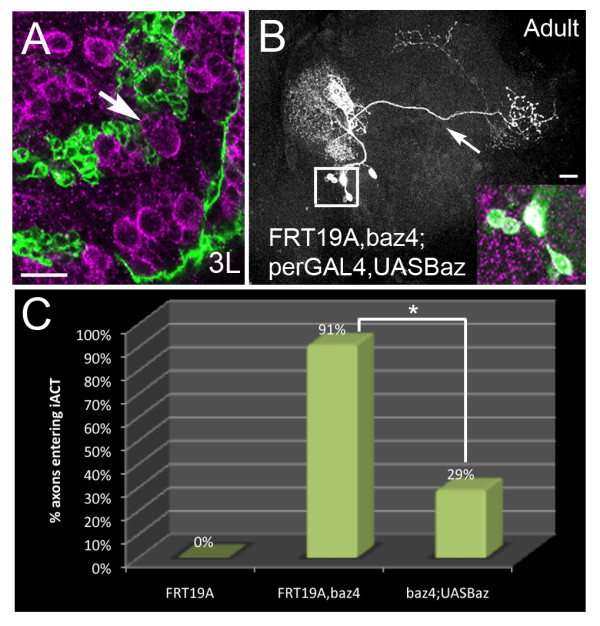
**Bazooka is an intrinsic requirement for axon pathway choice**. **(A) **Neuroblasts and newborn progeny in *period*-GAL4,UASGFP third instar larva brains labeled with anti-Bazooka (magenta). Newborn neurons were negative for green fluorescent protein (GFP)(arrow), indicating that *period-*GAL4 was not active in the ganglion mother cells (GMCs). **(B) **Mosaic analysis with a repressible cell marker (MARCM) *bazooka *loss-of-function (LOF) clone of the BAla1 lineage (green) re-expressing Bazooka protein (magenta) in mutant neurons (see inset). Note that the axon only entered the middle antennal cerebral tract (mACT) (arrow). **(C) **The percentage of axons entering the inner (i)ACT compared between control clones (0%, n = 28), *bazooka *LOF clones (91%, n = 11), and *bazooka *LOF clones re-expressing Bazooka protein only in vPNs (29%, n = 7). Asterisk indicates a significant decrease in the percentage of BAla1 LOF clones re-expressing Bazooka entering the iACT compared with the percentage of BAla1 LOF clones without Bazooka entering the iACT (*P *< 0.05). Scale bars: 15 μm

We next investigated if the guidance defects seen in the BAla1 *bazooka *LOF clones are due to a function of Bazooka in neuroblasts and GMCs, or to an intrinsic requirement of Bazooka in the neuron itself. *Period-*GAL4 was only active in post-mitotic neurons and not in the neuroblast or the transient GMCs (Figure 6A). To investigate Bazooka activity within post-mitotic neurons, we incorporated a UAS-Baz transgene into the FRT19A, *baz*^4^/FM7C;*period-*GAL4 MARCM line we used previously, and again induced clones at first instar. Because Bazooka protein is only expressed in the post-mitotic neurons of each clone, rescue of the misguidance phenotype would suggest that Bazooka regulates axon guidance in an autonomous manner, rather than at the level of neuroblast or GMC division. When Bazooka was re-expressed in the post-mitotic neurons of the BAla1 lineage (Figure6B, inset), significantly fewer clones entered the iACT (29%, n = 7, *P *< 0.05), indicating that re-expression of Bazooka in the differentiated neurons was sufficient to return the BAla1 trajectory to normal (Figure 6B,C). It is noteworthy that one of the rescued BAla1 SATs that did not enter the iACT had a trajectory similar to Bazooka overexpression (see below), in which the SAT initially entered a more lateral tract system, indicating that Bazooka was being expressed to baseline levels or greater. Also of note, the clone size of the rescue clones remained small, suggesting that Bazooka expression is required in the neuroblast or GMC for regulation of the *period *promoter in daughter neurons. Intrinsic rescue of the BAla1 guidance phenotype, taken together with the accumulation of ectopic Bazooka at the growth cone (Figure 3D), suggests that Bazooka autonomously (that is, through expression in the post-mitotic neuron) regulates axon guidance, possibly at the growth cone in the BAla1 lineage.

**Figure 7:**
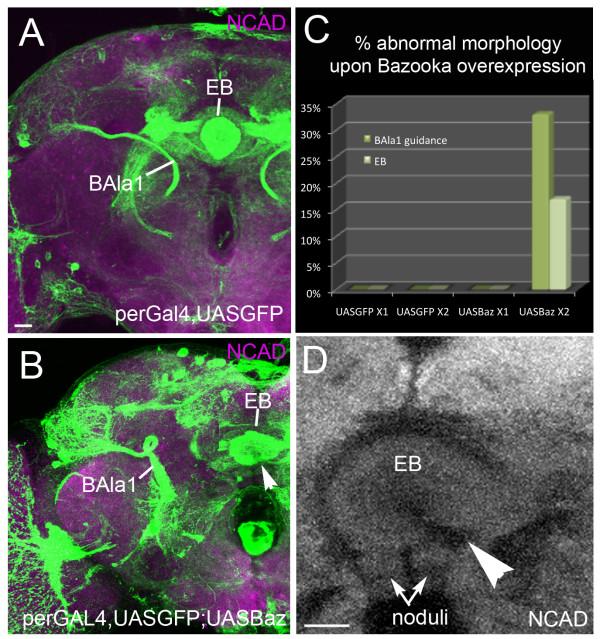
**Overexpression of Bazooka with period-GAL4 causes axon misguidance**. Confocal sections of adult brains at the level of the ellipsoid body (EB) are shown with homozygous **(A) ***period-*GAL4,UAS-GFP or **(B,D) **homozygous *period-*GAL4,UAS-GFP;UAS-Baz. **(D,E) **Note that the BAla1 lineage (ventral projection neurons) made a loop before terminating in the lateral horn, and the EB failed to close ventrally (arrowhead). **(C) **Graph comparing the percentage of abnormal lineage morphology in the presence of single copies of UAS-mcd8GFP (n = 7) and Bazooka (n = 3) versus double copies of UAS-mcd8GFP (n = 6) and Bazooka (n = 6). Note that only lineages containing double copies of UAS-Bazooka showed defects. Scale bars: 15 μm

If a loss of Bazooka leads to defective pathway control, does overexpression of Bazooka also affect BAla1 SAT trajectory? We next visualized the *period*-expressing lineages in both heterozygous and homozygous brains containing *period-*GAL4>UAS-GFP;UAS-Baz. Only brains containing two copies of UAS-Baz had aberrant trajectories, suggesting a dose-dependent effect of Bazooka on axon guidance (Figure 7C). In homozygous UAS-Baz brains, the BAla1 SAT presented early misguidance lateral to the typical ACT entrance, and subsequently looped around the peduncle in two of six hemispheres (Figure 7B). The DALv2 SAT failed to undergo ventral closure (Figure 7B,D). The presence of atypical projections in the BAla1 and DALv2 lineages upon Bazooka overexpression supports a role for Bazooka in axon growth and guidance.

### Par6 is required for BLD5 and DALv2 morphology, but not BAla1 or BAmv1

**Figure 8:**
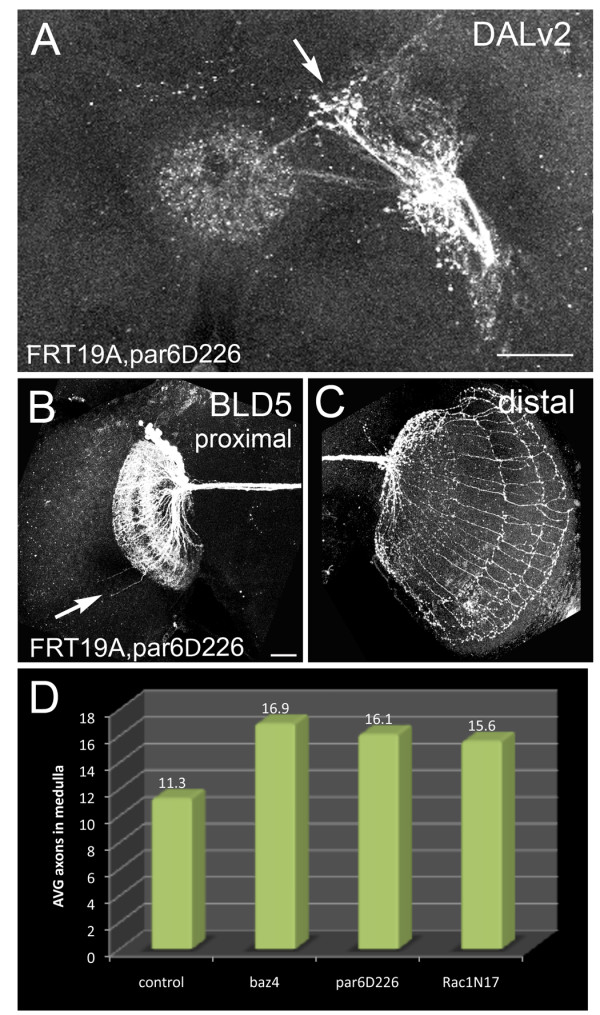
**Par6 regulates lineage morphology in DALv2 and BLD5**. Mosaic analysis with a repressible cell marker (MARCM) *par6 *loss-of-function (LOF) clones were generated at first instar and visualized in the adult via the period-GAL4 and atonal-GAL4 drivers. **(A) ***Par6 *LOF clone in the DALv2 lineage visualized with the period-GAL4 driver. Note ectopic proximal projections (arrow) compared with the stereotyped morphology (see Figure 4C). **(B,C) **Par6 LOF clone in the BLD5 lineage showing (B) an increase of proximal neurite projections into the ipsilateral medulla and (C) increased distal axons projecting into the contralateral medulla of the optic lobe. **(D) **A comparison of the average number of axons projecting into the contralateral optic medulla in each genotype is shown. Scale bar: 25 μm

To determine if Par6, another member of the Par complex, is required during axon pathway choice and the regulation of SAT morphology in the BAla1, DALv2, BAmv1 and BLD5 lineages, we generated *par6 *LOF clones using the *period*-GAL4 and *atonal*-GAL4 drivers. Neither the BAla1 (n = 8) nor the BAmv1 (n = 3) lineages showed an aberrant phenotype. However, *par6 *LOF clones in the DALv2 and BLD5 lineages resembled *bazooka *LOF phenotypes. In the DALv2 lineage, 43% of *par6 *LOF clones contained ectopic branching near the proximal domain (Figure 8A, n = 7) with a similar morphology to the *bazooka *LOF DALv2 clone (compare with Figure 4D). Furthermore, in the BLD5 lineage, *par6 *LOF clones had an average of 16 axons in the medulla (Figure 8B, n = 7) and an increased rate of proximal projections into the ipsilateral optic lobe, similar to the *bazooka *LOF BLD5 clones (compare with Figure 4H').

In both *bazooka *and *par6 *LOF BLD5 clones, the cell number was slightly larger than that of control clones. To verify that an increase in neuron number was not the basis for the increased number of projections, we plotted the cell number of each clone against the number of axons in the medulla. Although we did find an overall increase in clone size, comparing clones of only the same number (around 40 cells) still showed an increase in the number of distal extensions (see Additional file 3, Figure S3). Furthermore, if projection number into the medulla were directly proportional to cell number, we would expect the control clone containing approximately 60 cells to have an increased number of projections into the medulla, which it did not.

To determine if the increased number of distal extensions in the *bazooka *and *par6 *LOF clones was significant, we compared clones with a similar clone size (between 30 and 50 neurons) using a paired Student's *t*-test. The increased number of axons projecting into the medulla was significant for both the *bazooka *(*P *= 0.0018) and *par6 *(*P *= 0.0114) LOF clones.

## Discussion and Conclusions

In this study, we investigated the *in vivo *function of the Par-complex proteins, Par3/Baz and Par6, during neural development. Using MARCM and lineage-specific visualization, we investigated BAla1, BAmv1, DALV2 and BLD5 lineage morphologies in the absence of Bazooka and Par6 proteins. Our main method was to use a multilineage approach to explore the *in vivo *role of Par-complex proteins during axon growth and guidance. The mushroom body lineages have traditionally served as 'test' lineages when studying gene function in the *Drosophila *brain; however, the multilineage approach used in this study highlights the diverse functions of a protein complex between individual lineages.

### Bazooka lineage-dependent localization and function

Par3/Bazooka is a highly conserved gene found in both vertebrate and invertebrate species. In epithelial cells and neuroblasts, Par3/Bazooka localizes to the apical membrane, where it mediates spindle orientation, thereby establishing cellular polarity and assisting asymmetric cell division [9-13]. In post-mitotic hippocampal neurons, a cell type that no longer requires maintenance of asymmetric division, mammalian Par3 specifically localizes to the axon *in vitro *[4,5]. The dual use of a protein in polarized mitotic cells and in post-mitotic environments is an interesting area of exploration; however, little is known about the expression, localization or function of neuronal Par3 *in vivo*.

In the present study, we used both PD-type (BAla1 and BLD5) and C-type branching lineages (BAmv1 and DALv2) to study Bazooka localization and function. We found that the localization pattern of Baz:GFP in individual neural lineages correlated with *bazooka *LOF phenotypes. For example, larval BAla1 SATs localized Bazooka to the growth cone and exhibited axon-guidance defects in mutant *bazooka *clones. By contrast, BAmv1 and DALv2 SATs showed uniform distribution of ectopic Bazooka and subsequently had diffuse branching and neurite morphology defects along the length of the SAT upon Bazooka elimination.

It is important to note that we used single-copy Baz:GFP analysis when studying the localization of Bazooka in individual lineages. Subsequent experiments showed that two copies of UAS-Baz were required to induce morphologic changes in the BAla1 and DALv2 lineages (Figure 7). Therefore, it is likely that the gross morphology of the lineage was not disturbed while Bazooka localization was investigated. Taken together, our data suggest that Bazooka is used in a lineage-dependent manner depending on the ultimate morphology each lineage develops.

### Autonomous versus non-autonomous Bazooka function in neurons

The similarity between Bazooka localization to the growth cone of the BAla1 SAT and that of Par3 localization to the axon tip in cultured hippocampal cells is intriguing. Therefore, we focused on the BAla1 lineage to study the *in vivo *function of *bazooka *in neurons. Given that *bazooka *is expressed in neuroblasts and most, if not all, neurons (including the GMC precursors), the role of this gene in BAla1 SAT guidance has the potential to be either cell-autonomous or non-autonomous in the neuron. Based on the autonomous role of Bazooka during cultured hippocampal neuron development and its known functions during *in vitro *cell migration, we hypothesized that *bazooka *would also play an autonomous role *in vivo*. Several lines of evidence suggested that Bazooka is an autonomous factor regulating neural morphology. First, re-expression of Bazooka in the post-mitotic BAla1 neurons significantly restored BAla1 SAT guidance in mutant clones. Second, overexpression of Bazooka in only post-mitotic BAla1 neurons resulted in additional misguidance phenotypes, suggesting that tightly controlled balance of *bazooka *expression is required within the neuron. Third, *bazooka *was expressed within the post-mitotic neurons, and ectopic Bazooka:GFP entered the axon and accumulated at the growth cone, suggesting the presence of cell-autonomous machinery that actively shuttles Bazooka to specific sites in the BAla1 neurons.

### Bazooka regulates directional axon extension

The idea that polarity proteins can affect directional migration of the growth cone *in vivo *is consistent with cell migration studies *in vitro*. Par-complex members are required at the leading edge of cultured MDCK11, keratinoctyes and HeLa cells to regulate directional migration [17-19]. It is tempting to speculate that *bazooka *has a role in regulating cytoskeletal scaffolding during directional growth-cone guidance in *Drosophila *BAla1 neurons. In cultured rat hippocampal neurons, LIMK1, the kinase that phosphorylates the actin binding protein Cofilin, directs Par3 and Par6 to the growth cone and accelerates axon formation [32]. Furthermore, mammalian Par3 associates with LIMK2 during epithelial tight-junction formation [33]. Rac1 offers a tempting link between the Par complex, LIMK signaling and cytoskeletal rearrangement. Rac1 serves as an accessory to the Par complex [34-36], and can stimulate LIMK activity to regulate cytoskeletal rearrangement [37].

In contrast to cytoskeletal rearrangement, *bazooka *may instead function during signal transduction within the BAla1 neurons. Shrana and colleagues found that a balance between Wnt and fibroblast growth factor signaling controls axon retraction from the optic medulla in BLD5 neurons. The Wnt signal transducer Disheveled promotes axon differentiation in cultured hippocampal neurons by regulating aPKC, the binding partner for Par3 and Par6 [38]. Whether *bazooka *regulates the cellular machinery that allows for cytoskeletal rearrangement or serves as a mediator in signal transduction is an interesting area for further investigation.

### The Par complex acts in a lineage-dependent manner

Regardless of the mode of action *of bazooka*, its function does not seem to be equal in all lineages. The localization of ectopic Bazooka protein is not uniform across lineages, and *bazooka *null mutations generate SAT-dependent phenotypes, suggesting a differential requirement for Bazooka between individual SATs. Previous work [7] concluded that Par-complex proteins, including Baz, are not involved in establishing neuronal polarity, that is, distinction of dendritic versus axonal domains, in the lineages of the mushroom body. Our findings support this conclusion, as *bazooka *and *par6 *mutant clones still had discrete dendritic and axonal arborizations. The fact that loss of Baz and other Par-complex members apparently caused no structural abnormalities in the mushroom body suggests that these lineages do not require the Par complex for normal morphogenesis, which is not unexpected, given the differential requirement in other lineages analyzed in this study.

We found that *par6 *null mutations affected some SATs more severely than others, suggesting that the Par complex itself may act in a lineage-dependent manner. Because Bazooka has the ability to localize in some cells independently of the Par complex [13], it is possible that Bazooka has different modes of action depending on the morphologic needs of the neuron. We speculate that Bazooka acts independently from the Par complex during guidance of the BAla1 and BAmv1 SATs, where *par6 *LOF seems to have no effect; however, the similarity between *bazooka *and *par6 *LOF phenotypes in the DALv2 and BLD5 lineages points to the possibility that these neurons utilize the polarity proteins as a complex. Preliminary analysis of clones mutant for aPKC, randomly induced by the pan-neuronal driver elav-Gal4, yielded structural abnormalities in a number of lineages (SS and VH, unpublished data). The involvement of this and other Par-complex members in lineage morphogenesis awaits further study.

We have shown that Bazooka is expressed throughout the larval post-mitotic neurons, is transported to particular domains of the axon in a lineage-specific manner, and is required for branching regulation and axon guidance in correlation with its lineage-dependent localization patterns. BAla1 SAT guidance abnormalities in *bazooka *LOF clones could be rescued by post-mitotic specific re-expression of Bazooka protein, whereas overexpression of Bazooka in post-mitotic BAla1 neurons resulted in additional guidance phenotypes. Finally, we found that *par6 *LOF clones phenocopied *bazooka *LOF phenotypes in some, but not all lineages, suggesting a lineage-dependent use of the polarity proteins as a functioning complex.

Based on all the data, we speculate that bazooka acts in a cell-autonomous, lineage-dependent manner to control overall SAT morphology by mediating axon branching dynamics (in C-type lineages) or by steering axons into appropriate tract systems (in PD-type lineages) as they respond to targeting signals in the brain.

## Competing interests

The authors declare that they have no competing interests.

## Authors' contributions

SS participated in the design of the study, carried out the molecular genetic studies, performed the statistical analysis and drafted the manuscript. VH conceived of the study, participated in its design and helped to draft the manuscript. All authors read and approved the final manuscript.

## Supplementary Material

Additional file 1 **Figure S1. Developmental profile of period-GAL4 labeled lineages**. **(A'C) **BAla1, **(D-F) **DALv2 and **(G-I) **BAmv1. Mosaic analysis with a repressible cell marker (MARCM) clones were labeled with mcd8GFP at (left) late third instar, (center) mid-pupa and (right) adult stages. At each stage, the soma is labeled with arrowheads, proximal branches with arrows, and terminal branches with an asterisk. **(A,D,G) **Note that proximal branching is marked by a small tuft of filopodia at third instar (arrow). At third instar, single clones in the left hemisphere are shown. In pupal preparations, single clones are shown **(B) **in the BAla1 and **(E) **DALv2 lineages, and a clone in both hemispheres is shown in **(H) **the BAmv1 lineage. Finally, two clones are shown in the adult **(F) **DALv2 and **(I) **BAmv1 lineages. The neuropile is labeled with anti-*Drosophila *N-cadherin in all panels (purple). Abbreviations: AL = antennal lobe, CPL(m) = central posterolateral (medial) compartment, EB = ellipsoid body, FSB = fan-shaped body, IMP = inferior-medial protocerebrum, LH = lateral horn, LB = (lateral bulb), loBM = basomedial longitudinal tract system, MB = mushroom body,. Scale bars: 25 μm Click here for file

Additional file 2 **Figure S2. Antennal lobe glomerular innervation in mutant versus control BAla1 clones**. Percentage of clones of BAla1 neurons with strong dendritic projections in different glomeruli of the antennal lobe. All glomeruli are indicated on the *X*-axis. Red bars represent *bazooka *LOF clones, and blue bars represent control clones. The *Y*-axis represents the percentage of control or *bazooka *loss-of-function BAla1 clones with enhanced arbors in a given glomerulus. For example, wild-type clones had dense innervations of glomeruli DL2d or DM4 at a high frequency; the same glomeruli were targeted by *baz*-mutant clones, even at a lower frequency. In addition, there were a number of glomeruli (for example,, DA4, DL5) that, at low frequencies, were targeted by mutant clones Click here for file

Additional file 3 **Figure S3. Cell number versus distal projection number in BLD5 clones**. The *X*-axis indicates the number of cell bodies counted in the clone, and the *Y*-axis indicates the number of axons reaching into the contralateral optic lobe medulla in the respective clone. Note that at a count of 40 cells, the *baz*^4 ^and *par6*^D226 ^clones still had an increased number of distal projections compared with control clones Click here for file
