# Supplementary figures and images for: Bazooka mediates secondary axon morphology in Drosophila brain lineages
Source: Neural Dev. 2011 Apr 27;6:16. doi: 10.1186/1749-8104-6-16 (PMC3107162; doi:10.1186/1749-8104-6-16)

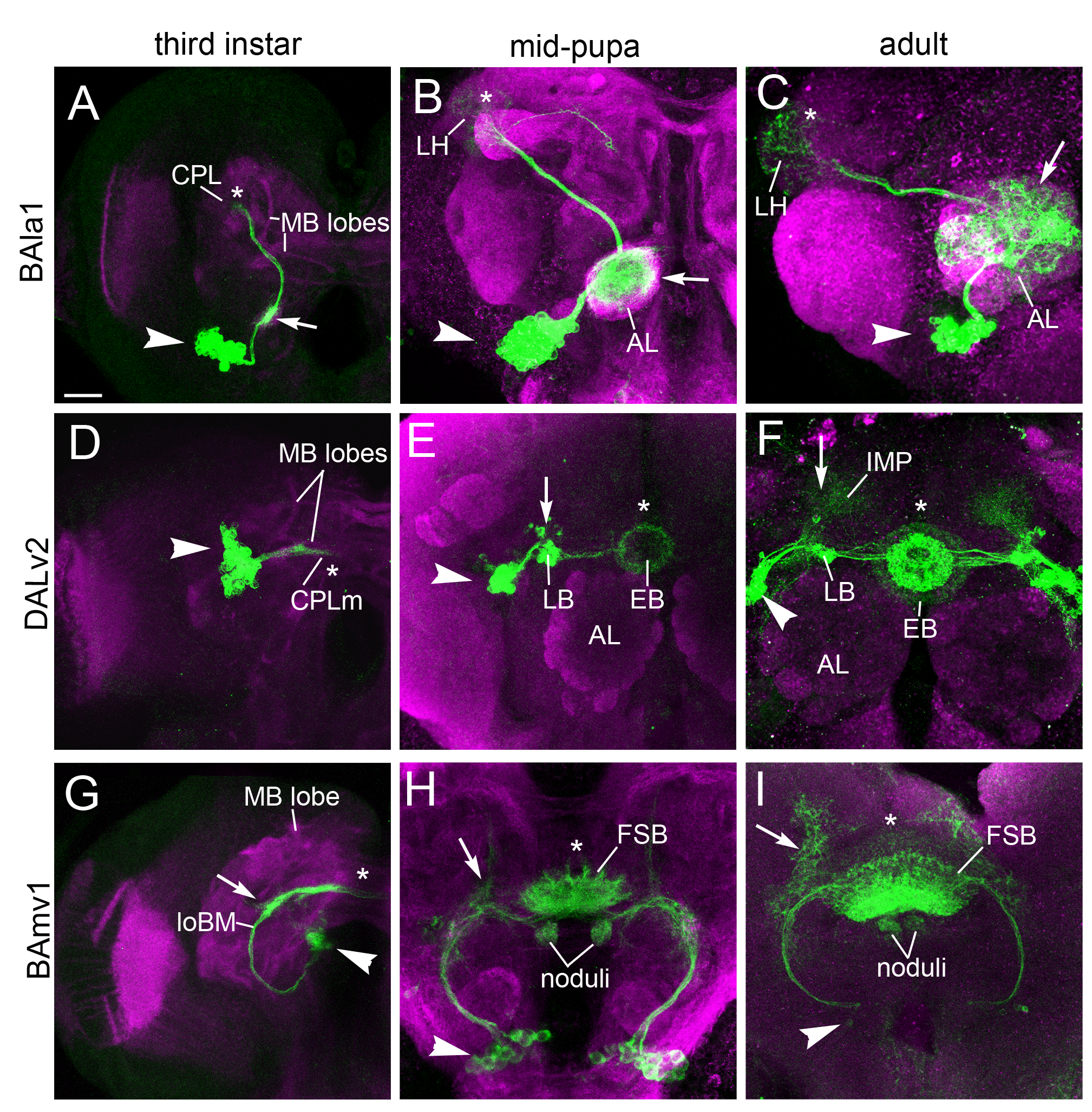

Supplement: Additional file 1 — Figure S1. Developmental profile of period-GAL4 labeled lineages. (A'C) BAla1, (D-F) DALv2 and (G-I) BAmv1. Mosaic analysis with a repressible cell marker (MARCM) clones were labeled with mcd8GFP at (left) late third instar, (center) mid-pupa and (right) adult stages. At each stage, the soma is labeled with arrowheads, proximal branches with arrows, and terminal branches with an asterisk. (A,D,G) Note that proximal branching is marked by a small tuft of filopodia at third instar (arrow). At third instar, single clones in the left hemisphere are shown. In pupal preparations, single clones are shown (B) in the BAla1 and (E) DALv2 lineages, and a clone in both hemispheres is shown in (H) the BAmv1 lineage. Finally, two clones are shown in the adult (F) DALv2 and (I) BAmv1 lineages. The neuropile is labeled with anti-Drosophila N-cadherin in all panels (purple). Abbreviations: AL = antennal lobe, CPL(m) = central posterolateral (medial) compartment, EB = ellipsoid body, FSB = fan-shaped body, IMP = inferior-medial protocerebrum, LH = lateral horn, LB = (lateral bulb), loBM = basomedial longitudinal tract system, MB = mushroom body,. Scale bars: 25 μm [file 1749-8104-6-16-S1.TIFF]

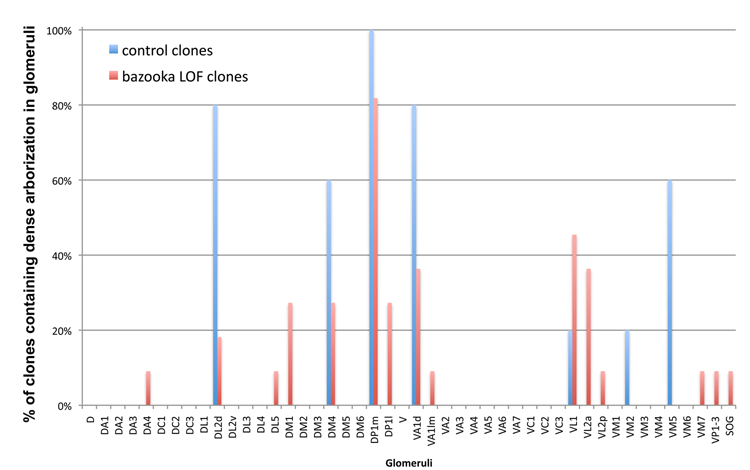

Supplement: Additional file 2 — Figure S2. Antennal lobe glomerular innervation in mutant versus control BAla1 clones. Percentage of clones of BAla1 neurons with strong dendritic projections in different glomeruli of the antennal lobe. All glomeruli are indicated on the X-axis. Red bars represent bazooka LOF clones, and blue bars represent control clones. The Y-axis represents the percentage of control or bazooka loss-of-function BAla1 clones with enhanced arbors in a given glomerulus. For example, wild-type clones had dense innervations of glomeruli DL2d or DM4 at a high frequency; the same glomeruli were targeted by baz-mutant clones, even at a lower frequency. In addition, there were a number of glomeruli (for example,, DA4, DL5) that, at low frequencies, were targeted by mutant clones [file 1749-8104-6-16-S2.TIFF]

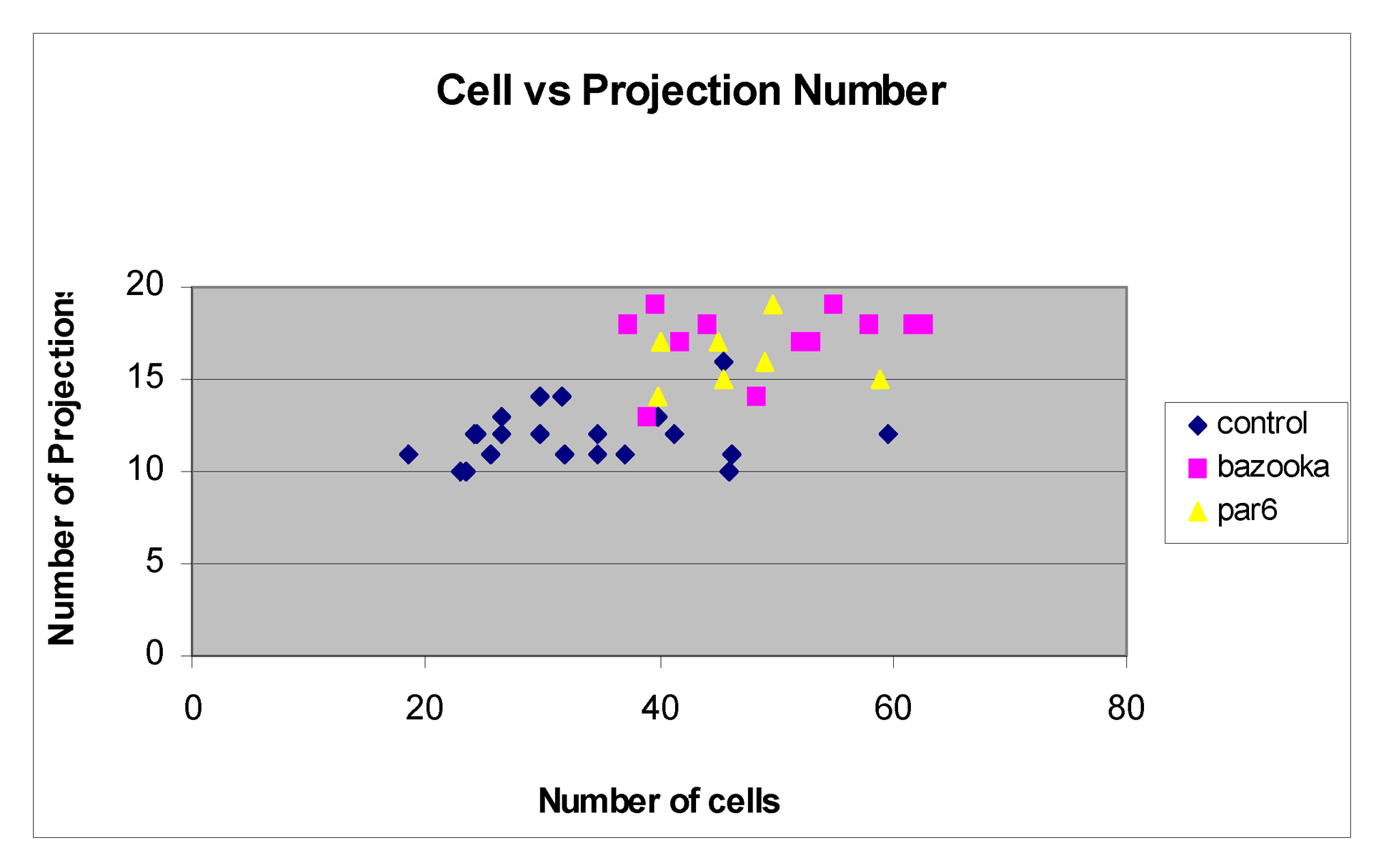

Supplement: Additional file 3 — Figure S3. Cell number versus distal projection number in BLD5 clones. The X-axis indicates the number of cell bodies counted in the clone, and the Y-axis indicates the number of axons reaching into the contralateral optic lobe medulla in the respective clone. Note that at a count of 40 cells, the baz4 and par6D226 clones still had an increased number of distal projections compared with control clones [file 1749-8104-6-16-S3.TIFF]
